# A retrospective chart review of central centrifugal cicatricial alopecia patients at a single urban institution

**DOI:** 10.1016/j.jdin.2023.07.014

**Published:** 2023-08-09

**Authors:** Eliza Balazic, Anna Chen, Hailey Konisky, Kelly Hawkins, Janet Choi, Nour Mhaimeed, Chesahna Kindred, Kseniya Kobets

**Affiliations:** a Division of Dermatology, Department of Medicine, Albert Einstein College of Medicine, Montefiore Medical Center, Bronx, New York; b Eastern Virginia Medical School, Norfolk, Virginia; c Kindred Hair and Skin Center, Columbia, Maryland; d Department of Dermatology, Howard University, Washington, District of Columbia

**Keywords:** alopecia, central centrifugal cicatricial alopecia, general dermatology, medical dermatology, skin of color

*To the Editor:*

Central centrifugal cicatricial alopecia (CCCA) is a primary scarring alopecia that affects primarily Black women and begins with hair loss at the vertex or crown of the scalp.^1^ In 2010, Shah and Alexis conducted a retrospective chart review of 69 patients focusing on clinical features and hair care practices.^2^ No singular International Classification of Disease (ICD)-10 code for CCCA exists, which makes larger epidemiologic studies difficult. This study aims to help bridge that gap and characterize CCCA patients by providing insight into the demographics, characteristics, and treatment.

A retrospective medical record review was conducted at Montefiore Medical Center from July 2015 to March 2022 of patients diagnosed clinically with CCCA. All charts with an ICD-10 code for scarring alopecia, including subtypes, other alopecia, and general alopecia were manually screened as no singular ICD-10 code for CCCA exists.

One hundred eighty-five unique patients with CCCA were identified from July 2015 to March 2022. Patient demographic information and select medical history were collected (Table I). All patients were female with 82.7% identifying as Black or African American. Other races with a CCCA diagnosis included other (11.4%), unknown race (4.3%), and White (1.6%). The mean age at presentation was 53.3 years. The mean time of onset was 3.7 years prior to the initial presentation.

**Table I:** Characteristics

Characteristic			Percentage (raw count)
Demographics	Sex	Female	100% (185)
		Male	0% (185)
	Race	Black or African American	82.7% (153)
		White	1.6% (3)
		Other	11.4% (21)
		Unknown	4.3% (8)
	Ethnicity	Hispanic	5.4% (10)
		Non-Hispanic	84.9% (157)
		Not specified	9.7% (18)
	Age, y	53.7	Minimum 17, maximum 93
Medical history	Smoker	Current smoker	4.8% (9)
		Former smoker	16.2% (30)
		Never smoker	75.1% (139)
		Unknown smoking history	2.7% (5)
	Diabetes mellitus type II	23.7% (44)	
	Hyperlipidemia	35.1% (65)	
	Thyroid condition	Hyperthyroidism	0.5% (1)
		Hypothyroidism	3.2% (6)
		Other thyroid condition	2.2% (4)
	Hypertension	51.3% (95)	

Charts were evaluated at first presentation (Table II). For 69.7% of patients, hair loss was the chief complaint at the first visit. The most reported symptom was itching in 23.3% of patients. However, most charts reported no associated symptoms. Around a third of patients had a reported history of chemical treatments. Additionally, 79.5% specified hair loss at the vertex or crown of the head. Biopsy results were available for 11.4% of patients at any time. Treatment was administered to 85.9% of patients, with topical medications being the most prevalent medication modality including topical minoxidil (38.4%), topical fluocinonide (21.4%), and ketoconazole shampoo (19.5%). Intralesional triamcinolone injections (alone, with topicals, or with topicals and oral medication) were administered to 20.1% of patients.

**Table II:** First visit

Hair loss as chief complaint	Yes	69.7% (129)	No	30.3% (56)
Hair loss duration	42.2% (78) specified	3.7 y		
Any previous treatment	36.2% (67)			
Itching	Yes	23.3% (43)	No	23.7% (44)
			Not specified	53.0% (98)
Burning	Yes	2.1% (4)	No	24.9% (46)
			Not specified	72.9% (135)
Tenderness	Yes	4.3% (8)	No	24.3% (45)
			Not specified	71.4% (132)
Reported hair practices	History of chemical styling including perms, chemical relaxers	Yes	30.2% (56)	
	History of tight hairstyling practices including braids and sewn-in hair pieces	Yes	26.5% (49)	
	History of hot comb use	Yes	3.2% (6)	
Location of hair loss	Vertex	79.5% (147)	Temporal	9.7% (18)
	Frontal	20.5% (38)		
Biopsy taken	At first visit	3.8% (7)	At any visit	11.4% (21)
Treatment given	85.9% (159)			
Treatment type	Topical only	69.8% (111)	Topicals and oral	8.2% (13)
	Injections only	1.3% (2)	Injections and orals	0% (0)
	Oral only	1.9% (3)	Topicals, injections, and oral	7.5% (12)
	Topicals and injections	11.3% (18)		
Specific treatments∗	Intralesional kenalog	20.1% (32)	Tacrolimus	2.5% (14)
	Minoxidil 5%	38.4% (61)	Betamethasone	14.5% (23)
	Topical clobetasol	17.6% (28)	Halobetasol	6.3% (10)
	Ketoconazole shampoo	19.5% (31)	Fluocinolone	9.4% (15)
	Fluocinonide	21.4% (34)	Triamcinolone acetonide ointment	6.3% (10)
	Doxycycline	9.4% (15)		
Follow-up recommended	Specified	36.8% (185)	Time	2.7 mo

∗ Only included if there were 10 or more. Other treatments included selenium sulfide (8), biotin (6), spironolactone (3), oral minoxidil, oral finasteride, dexamethasone, mometasone, and pimecrolimus (1).

The study limitations include those inherent to retrospective studies as it only represents the information available in the medical record at a singular medical center and represents a singular patient population diagnosed clinically with no control group. Although CCCA requires a biopsy for definitive diagnosis often it could be diagnosed clinically in classic scenarios based on physical examination including clinical^3^ and dermatoscopy findings^4^, which may explain the low rates of biopsy in this cohort. There are no current published studies on the rates of biopsy in scarring alopecias.

This study reaffirms CCCA as a disease that primarily affects Black or African American females. The disease presents in middle age; however, this does not necessitate that it begins in middle age. CCCA can present silently with most patients reporting no symptoms, which could lead to delayed intervention. In addition, the hair loss could present in a site not easily visualized by the patient. CCCA needs its own unique ICD-10 code to make larger epidemiologic studies possible.

## Conflicts of interest

Dr Kindred has served as an advisory board and speaker’s bureau member for Lilly and Sun Pharmaceutical, as a speaker and advisory board member for UCB and Regeneron, as a board member for Aerolase, as a speaker for Novartis, as a speaker’s bureau member for Nutrafol, as an editor for Cutis and JNMA, as a web content reviewer for AAD, as a steering committee and Skin of Color advisory board for Janssen, and as a consultant for AbbVie and Pfizer. Authors Balazie, Chen, Konisky, Hawkins, Choi, and Mhaimeed and Dr Kobets have no conflicts of interest to declare.
